# Effects of supplementing docosahexaenoic acid-rich microalgae and antioxidants on beef longissimus lumborum steak color stability and sensory characteristics^1,2^

**DOI:** 10.1093/tas/txaa135

**Published:** 2020-07-18

**Authors:** Kelsey J Phelps, James S Drouillard, Travis G O’Quinn, Terry A Houser, John M Gonzalez

**Affiliations:** 1 Cargill Protein, Wichita, KS; 2 Department of Animal Sciences and Industry, Kansas State University, Manhattan, KS; 3 Department of Animal Science, Iowa State University, Ames, IA; 4 Department of Animal and Dairy Science, University of Georgia, Athens, GA

**Keywords:** antioxidant, beef, color, microalgae, palatability

## Abstract

The objective of this study was to determine the effects of four microalgae and antioxidant feeding regimens on beef longissimus lumborum color stability and palatability. Steers were blocked by weight and randomly assigned to one of four dietary treatments fed during a 45-d feeding period. Treatments (*n* = 10 per treatment) consisted of a control diet (CON) and control diet plus 100 g∙steer^−1^∙d^−1^ microalgae (ALGAE), ALGAE plus antioxidants (103 IU/d vitamin E and Sel-Plex) fed throughout feeding (AOX), and AOX fed for the final 10 d of finishing (LATE). The longissimus lumborum muscle was removed, aged for 14 d, and fabricated into steaks for objective and subjective color and palatability analyses. There were treatment × day of display interactions for a* value and steak surface metmyoglobin percentage (*P* < 0.01). There were no treatment differences through day 4 of display for a* value (*P* > 0.16) and day 5 of display for surface metmyoglobin (*P* > 0.10). By day 10 of display, ALGAE steaks had a smaller a* value than all other treatments (*P* < 0.01). Steaks from AOX steers had a greater (*P* < 0.01) a* value than CON steaks, whereas both a* values did not differ from LATE steaks (*P* > 0.19). By the end of display, ALGAE steaks had more metmyoglobin than the other treatments (*P* < 0.01). Steaks from AOX steers had less metmyoglobin than CON and LATE steaks (*P* < 0.04), which did not differ (*P* > 0.25). Treatment did not affect trained panel ratings (*P* > 0.15); however, treatment did affect (*P* < 0.01) off-flavor intensity. Steaks from ALGAE and AOX steers had greater off-flavor ratings than CON steaks (*P* < 0.03), but did not differ (*P* = 0.10). Steaks from LATE steers did not differ in off-flavor ratings from the other treatments (*P* > 0.07). Use of antioxidants improved color stability of steaks from microalgae fed steers; however, panelists still detected off-flavors.

## INTRODUCTION

Beef, one of the major meat protein sources in the United States, is regarded as having relatively high concentrations of saturated fatty acids compared with other protein sources, especially fish. Because of this reputation, scientists and consumers perceive beef as being unhealthy (Luo and Huang, 2012). Omega-3 fatty acids are a family of polyunsaturated fatty acid (PUFA) that provide numerous health benefits, including reduced risks of cardiovascular disease, type-2 diabetes, and cancer (Ruxton et al., 2004; Calder, 2014) and may also be used as therapy to treat personality disorders such as bipolar disorder (Saunders et al., 2015). In particular, the long-chain omega-3 fatty acids eicosapentaenoic acid (EPA) and docosahexaenoic acid (DHA) are the most functionally active within the body (Calder, 2014). Because most Americans do not consume adequate amounts of fatty fish, research has focused on manipulating the fatty acid profile of beef to achieve greater amounts of omega-3 fatty acids. The main strategy used to manipulate the fatty acid profile of beef is through the dietary supplementation of oilseeds, plant oils, fish oil, marine algae, and fat supplements (Woods and Fearon, 2009).

Multiple ruminant studies reported that ruminally protected microalgae increased DHA and EPA content by a maximum of 575% (Cooper et al., 2004; Nute et al., 2007). Including these studies, the literature exhibited that feeding aquatic sources of fatty acids elicited adverse effects on flavor and meat color during display (Vatansever et al., 2000; Wistuba et al., 2006; Daly et al., 2007). In prior performed studies, Phelps et al. (2016a,b) demonstrated feeding increasing levels of microalgae (*Schizochytrium limacinum* CCAP 4087/2) to finishing heifers increased steak and ground beef DHA and EPA content by up to 850%; however, these diets drastically accelerated lipid oxidation and color deterioration, while also increasing product off-flavors. Although the increased meat omega-3 content was the greatest reported in the literature, the negative effects on color stability and palatability would prevent this feeding strategy from being widely accepted. Therefore, alternative feeding strategies using this source of microalgae, such as reduced feeding time or antioxidant supplementation, require further exploration.

Numerous studies reported feeding elevated levels of the antioxidant vitamin E during the finishing phase help mitigate lipid oxidation and color deterioration during display (Yang et al., 2002; Gobert et al., 2010). In addition to vitamin E, selenium has also been considered for use as a muscle antioxidant. The current data suggested that selenium supplementation does not affect color (O’Grady et al., 2001; Taylor et al., 2008; Jose et al., 2010); however, source of selenium (organic vs. inorganic) may influence its usefulness in muscle (Rossi et al., 2015). The objective of this study was to evaluate the effects of microalgae (*S. limacinum* CCAP 4087/2) supplementation period , and vitamin E and selenium-yeast supplementation to feedlot steers on display color and palatability of beef longissimus lumborum (LL) steaks.

## MATERIALS AND METHODS

All experimental procedures were approved by the Kansas State University (KSU) Institutional Animal Care and Use Committee and the KSU Institutional Review Board for use of human subjects in sensory panel evaluations.

### Animals

Black-hided crossbred feedlot steers (638 ± 29 kg initial body weight) were housed in uncovered, dirt-surfaced pens (10 × 19.8 m; 4 pens; 10 steers per pen) that provided 10 m of linear bunk space and were equipped with watering fountains between adjacent pens. Prior to the start of the experiment, animals were weighed, ordered, and blocked into groups of four by initial body weight, and assigned within each four head strata to one of four dietary treatments (*n* = 10 per treatment; Table 1). Dietary treatments were control diet (CON) and control diet plus 100 g∙steer^−1^∙d^−1^ supplemental microalgae meal (ALGAE; Alltech Inc., Nicholasville, KY), 100 g∙steer^−1^∙d^−1^ supplemental microalgae meal plus antioxidants (103 IU/d vitamin E and Sel-Plex; Alltech Inc.) fed throughout feeding (AOX), and 100 g∙steer^−1^∙d^−1^ supplemental microalgae meal plus antioxidants fed for the final 10 d of finishing (LATE). All experimental diets were fed for 45 d prior to harvest.

**Table 1.** Calculated diets (dry matter basis) of steers fed four dietary microalgae meal and antioxidant feeding strategies

	Treatment^1^			
Ingredient, %	CON	ALGAE	AOX	LATE
Steam-flaked corn	46.72	45.97	45.91	45.91
Wet-corn gluten feed	30.00	30.00	30.00	30.00
Corn silage	10.00	10.00	10.00	10.00
Ground flax	10.00	10.00	10.00	10.00
Monensin and tylosin premix^2^	1.35	1.35	1.35	1.35
Mineral/vitamin supplement^3^	0.05	0.05	—	—
Mineral/vitamin supplement—no selenium selenite^4^	—	—	0.01	0.01
Microalgae meal supplement^5^	—	0.75	0.69	0.69
Ground limestone	1.59	1.59	1.59	1.59
Salt	0.25	0.25	0.25	0.25
Vitamin A^6^, 30,000 IU/g	0.007	0.007	0.007	0.007
Vitamin E^6^, 44,092 IU/g	0.03	0.03	0.14	0.14

^1^Diets consisted of a control diet (CON) or control diet plus 100 g∙steer^−1^∙d^−1^ supplemental microalgae meal (ALGAE; Alltech Inc., Nicholasville, KY), 100 g∙steer^−1^∙d^−1^ supplemental microalgae meal plus antioxidants (103 IU/d vitamin E and Sel-Plex; Alltech Inc.) fed throughout feeding (AOX), and 100 g∙steer^−1^∙d^−1^ supplemental microalgae meal plus antioxidants fed for the final 10 d of finishing (LATE). ^2^Provided 300 mg∙steer^−1^∙d^−1^ of monensin and 90 mg∙steer^−1^∙d^−1^ of tylosin (Elanco Animal Health, Greenfield, IN) in a ground corn carrier. ^3^Diets included CuSO_4_ to prove 10 mg/kg Cu, CoCO_3_ to prove 0.15 mg/kg Co, ethylenediamine dihyriodide to prove 0.5 mg/kg I, MnSO_4_ to prove 60 mg/kg Mn, Na_2_SeO_3_ to provide 0.3 mg/kg Se, and ZnSO_4_ to provide 60 mg/kg Zn in the total mixed ration on a dry matter (DM) basis. ^4^Diets included CuSO_4_ to prove 10 mg/kg Cu, CoCO_3_ to prove 0.15 mg/kg Co, ethylenediamine dihyriodide to prove 0.5 mg/kg I, MnSO_4_ to prove 60 mg/kg Mn, ZnSO_4_ to provide 60 mg/kg Zn, and an organic yeast (Sel-Plex, Alltech Inc., Nicholasville, KY) to provide 0.3 mg/kg Se in the total mixed ration on a DM basis.

^5^Provided 100 g∙steer^−1^∙d^−1^ of microalgae (*Schizochytrium limacinum* CCAP 4067/2, Alltech Inc.). ^6^Added 2,205 IU/kg vitamin A to all diets, 22 IU/kg vitamin E to CON and ALGAE diets, and 103 IU/kg vitamin E to AOX and LATE diets.

### Carcass Data Collection, Loin Collection, and Processing

Steers were transported 262 km to a commercial abattoir for harvest (Creekstone Farms; Arkansas City, KS). Immediately following harvest, hot carcass weight and incidence of liver abscesses data were collected. After a 48-h chilling period, standard carcass data were collected by trained university personnel, and strip loins (Institutional Meat Purchase Specifications #180; NAMP, 2010) were removed from the right side of each carcass, vacuum packaged, and transported to the KSU Meat Laboratory (Manhattan, KS). Loins were stored at 2 ± 1 °C until day-14 postmortem.

After storage, packages were opened, and four 2.54-cm-thick steaks were fabricated from the anterior to the posterior. Steak 1 was utilized for day 0 of display metmyoglobin-reducing ability (MRA) and oxygen consumption (OCR) analyses, steak 2 was used for day 5 of display MRA and OCR analyses, and steak 3 was used for daily objective color measurements and day 10 of display MRA and OCR analyses. Steak 4 was vacuum packaged, frozen at −40 °C, and ultimately utilized for trained sensory panel analysis.

### Simulated Retail Display

Steaks used for day-5 and day-10 simulated retail display were placed on 17S polystyrene foam trays with a UltraZap-40 absorbent pad (Paper-Pak Industries Inc., La Verne, CA) and overwrapped with polyvinyl chloride film (AEP Industries, South Hackensack, NJ) possessing an oxygen transmission rate of 23,250 cm^−3^·m^−2^·24 h^−1^ at 23 °C and 0% relative humidity. With respect to anatomical location of the beef animal, steaks were orientated on trays with the posterior end facing up and medial portion on the left. Steaks were divided among two coffin-style retail cases (Model DMF 8; Tyler Refrigeration Corporation, Niles, MI), ensuring treatments were represented equally in each case. Steaks were displayed under fluorescent lights (32 W Del-Warm White 3000K; Philips Lighting Company, Somerset, NJ) that emitted a constant 24-h case average intensity of 2,190 ± 34 lx. Cases were set to operate at 2 ± 1 °C and defrosted every 12 h at 13 °C for 30 min. Case temperatures were monitored using a Thermochron iButton (Maxim Integrated Products, Sunnyvale, CA) and average recorded temperature of the cases was 1.94 ± 0.95 °C. Every 12 h, steaks were rotated within their cases from left to right and front to back to account for minor variations in temperature and light intensity within the cases.

Every 24 h on day-10 steaks, surface color readings including CIE L*a*b* and reflectance from 400 to 700 nm were collected at three locations on each steak using a Hunter Lab Miniscan EZ spectophotometer (Illuminant A, 2.54-cm-diameter aperture, 10° observer; Hunter Associates Laboratory, Reston, VA). Surface reflectance values at 473, 525, 572, and 700 nm (473 and 572 nm were calculated) were used to calculate surface percentages of metmyoglobin and oxymyoglobin using equations from Krzywicki (1979) as published in the American Meat Science Association’s (AMSA) Meat Color Measurement Guidelines (AMSA, 2012). Values from the three scans were averaged to yield a steak average.

In addition to objective color data collection, subjective redness and surface discoloration were assessed by a trained visual panel daily. A total of 12 panelists, who were initially screened using the Farsnworth-Munsell 100 Hue Color Vision Test, were oriented to redness and discoloration evaluation over four training sessions. Utilizing continuous 100-mm line scales on paper ballots, panelists evaluated redness using six anchors points where 0 mm = 1 or light-pinkish red and 100 mm = 6 or very dark red (Van Bibber-Krueger et al., 2020), with anchors spaced every 20 mm. Additionally, panelists assessed surface discoloration as a percentage of the longissimus muscle area also on 100-mm continuous line scales with anchors at 0 = no discoloration and 100 = 100% discoloration. Eight panelists participated in panel daily, and panelist responses (distance from 0 mm for each attribute) were measured using a DrawingBoard VI (GTCO Calcomp, Turning Technologies, Scottsdale, AZ) and TabletWorks Software (Version 10.10; GTCO Calcomp, Turning Technologies).

### Metmyoglobin-Reducing Ability and Oxygen Consumption Rate

At the time of analysis (days 0, 5, and 10), each steak was split laterally into equal 1.27-cm-thick steaks. The display-side portion was utilized for MRA analysis, and the freshly cut portion was utilized for OCR. The procedures from Phelps et al. (2014) were followed for MRA. Briefly, steaks were cut into three locations to represent the entire steak. Each section was placed in a beaker and oxidized in 100 mL of 0.3% sodium nitrite at 25 ± 2 °C for 20 min. Following incubation, samples were blotted of excess solution and vacuum packaged in 25.4 × 30.5 cm vacuum bags (3 mil standard barrier, Prime Source Vacuum Pouches; Bunzl Processor Division, Koch Supplies, Kansas City, MO) that possessed an oxygen transmission rate of 4.3 cm^3^∙100 cm^−2^∙24 h^−1^ at 23 °C and 65% relative humidity. Reflectance measurements were taken from 400 to 700 nm at 0 and 2 h using a Hunter Lab Miniscan EZ spectrophotometer (Illuminant A, 2.54-cm-diameter aperture, 10° observer; Hunter Associates Laboratory). Spectral readings at 525, 572, and 700 nm from the three locations were averaged and used to calculate metmyoglobin percentages using equations described from Krzywicki (1979) as published in the AMSA Meat Color Measurement Guidelines (AMSA, 2012). Metmyoglobin-reducing ability was calculated as follows: (observed decreased in metmyoglobin concentration/initial metmyoglobin concentration) × 100.

Oxygen consumption was conducted as described in the AMSA Meat Color Measurement Guidelines (AMSA, 2012). The bottom portion of the steak fabricated for OCR was allowed to oxygenate for 2 h at 4 °C, covered with polyvinyl chloride film (AEP Industries) possessing an oxygen transmission rate of 23,250 cm^−3^·m^−2^·24 h^−1^ at 23 °C and 0% relative humidity. Following oxygenation, steaks were cut into the three sublocations, vacuum packaged in 25.4 × 30.5 cm vacuum bags described previously, and incubated at 25 °C for 40 min. Reflectance measurements from 400 to 700 nm were collected immediately following packaging and after 40 min of incubation. Spectral readings at 473, 525, 572, and 700 nm were averaged across the three locations to calculate surface oxymyoglobin percentage using equations described earlier. Oxymyoglobin consumption was calculated as follows: (observed decreased in oxymyoglobin concentration/initial metmyoglobin concentration) × 100.

### Sensory Analysis

Sensory analyses were conducted according to the procedures outlined in the AMSA Research Guidelines for Cookery, Sensory Evaluation, and Instrumental Tenderness Measurements of Meat (AMSA, 2015), with training and anchors similar to those described by Lucherk et al. (2016) and Vierck et al. (2018). Twenty-four hours prior to cooking, steaks were thawed on trays at 2.7 ± 0.9 °C. Before cooking, a thermocouple (30-gauge copper and constantan; Omega Engineering, Stamford, CT) was inserted into the geometric center of each steak. Steaks were cooked on clam-style grills (Cuisinart Griddler; Cuisinart, Stamford, CT) set to 177 °C and removed from grills at 70 °C. After cooking, steaks were cut into 1.27 × 1.27 × 2.54 cm cubes and presented to an eight-member-trained sensory panel.

Panelists were selected from a larger pool of candidates trained according the Research Guidelines for Cookery, Sensory Evaluation, and Instrumental Tenderness Measurements of Meat (AMSA, 2015). Selected panelists were oriented to strip loin steak evaluation over four training sessions prior to the initiation of panels. At each panel, panelists were seated at individual cubicles in a room designed for subjective sensory panel analysis and were presented two cubes from each of eight steaks (*n* = 2 per treatment/panel). Panelists evaluated the two cubes for initial juiciness, sustained juiciness, myofibrillar tenderness, connective tissue amount, overall tenderness, beef-flavor identity, beef-flavor intensity, and off-flavor intensity using 100-mm continuous line scales on paper ballots. The 0-mm anchors were as follows: extremely dry, extremely dry, extremely tough, none, extremely tough, extremely unbeef-life, extremely bland, and extremely bland. Conversely, the 100-mm anchors were as follows: extremely juicy, extremely juicy, extremely tender, abundant, extremely tender, extremely beef-like, extremely intense, and extremely intense. Also, there were midpoint (50%) anchors for the initial juiciness, sustained juiciness, myofibrillar tenderness, overall tenderness, and beef identity scales. These anchors were labeled as neither dry nor juicy, neither dry nor juicy, neither tough nor tender, neither tough nor tender, and neither unbeef-like nor beef-like. A total of five separate panels were conducted to analyze all the samples.

Panelist responses were measured using a DrawingBoard VI (GTCO Calcomp, Turning Technologies, Scottsdale, AZ) and TabletWorks Software (Version 10.10; GTCO Calcomp, Turning Technologies). Data are expressed as the distance on the scale of 0–100 mm.

### Statistical Analysis

Data were analyzed as a randomized complete block design using the GLIMMIX procedure of SAS 9.4 (SAS Inst. Inc., Cary, NC), with animal as the experimental unit and steak as the observational unit. Treatment served as the fixed effect, and weight block served as the random effect. For simulated retail display data, data were analyzed as a randomized complete block design with repeated measures. Treatment, day of display, and their interaction served as fixed effects, and weight block served as the random effect. Day of display served as the repeated measure with steak (observational unit) as the subject and compound symmetry as the covariance structure because observation days were equally spaced apart. Differences were considered significant at *P* ≤ 0.05.

## RESULTS

### Carcass Characteristics

Treatment affected final body weight, hot carcass weight, and ribeye area (*P* < 0.04), but did not affect other measures (*P* > 0.08; Table 2). Steers from the ALGAE treatment had a greater final body weight than AOX and LATE steers (*P* < 0.01), but these two treatments did not differ (*P* = 0.83) from each other. The final body weight of CON steers did not differ from all other treatments (*P* > 0.06). Carcasses from CON and ALGAE steers had greater hot carcass weights than AOX carcasses (*P* < 0.01), but these treatments did not differ (*P* = 0.87) from each other. Carcass from LATE steers did not differ in hot carcass weight compared with the other treatments (*P* > 0.07). Carcasses from CON steers had a greater ribeye area than all other treatments (*P* < 0.03), whereas all three treatments did not differ from each other (*P* > 0.72).

**Table 2.** Body weights and carcass characteristics of steers fed four dietary microalgae meal and antioxidant feeding strategies

	Treatment^1^					
	CON	ALGAE	AOX	LATE		
	*n* = 10	*n* = 10	*n* = 10	*n* = 10	SEM	*P*-value
Body weight, kg						
Initial	638.3	642.1	636.6	640.5	9.5	0.39
Final	727.5^a,b^	733.8^a^	715.2^b^	713.7^b^	10.1	0.02
Hot carcass weight, kg	465.6^a^	466.5^a^	451.0^b^	456.8^a,b^	6.9	0.01
Dressing percentage	64.0	63.6	63.0	64.0	0.3	0.08
Ribeye area, cm^2^	100.7^a^	93.1^b^	91.9^b^	92.2^b^	2.5	0.04
Subcutaneous backfat, cm	1.9	2.0	1.9	1.8	0.1	0.59
Marbling score^2^	630	573	599	591	29	0.40
Yield grade	3.7	4.2	4.0	3.9	0.2	0.38

^a,b^Means within a row with different superscripts differ (*P* < 0.05). ^1^Diets consisted of a control diet (CON) or control diet plus 100 g∙steer^−1^∙d^−1^ supplemental microalgae meal (ALGAE; Alltech Inc., Nicholasville, KY), 100 g∙steer^−1^∙d^−1^ supplemental microalgae meal plus antioxidants (103 IU/d vitamin E and Sel-Plex; Alltech Inc.) fed throughout feeding (AOX), and 100 g∙steer^−1^∙d^−1^ supplemental microalgae meal plus antioxidants fed for the final 10 d of finishing (LATE). ^2^Marbling score: 100 = practically devoid; 200 = traces; 300 = slight; 400 = small; 500 = modest; 600 = moderate; 700 = slightly abundant.

### Simulated Retail Display Color

**Figure 1.**
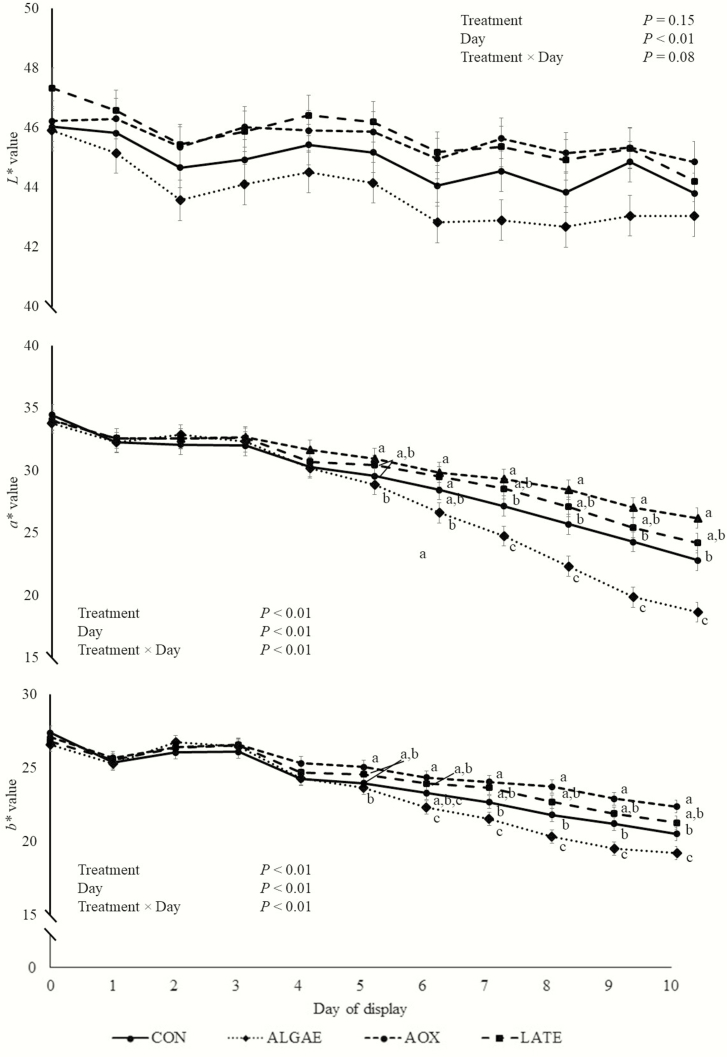
Longissimus lumborum steak L* (lightness: 0 = black, 100 = white), a* (redness: −60 = green, 60 = red), and b* values (blueness: −60 = blue, 60 = yellow) from steers fed four dietary microalgae meal and antioxidant feeding strategies (*n* = 10 per treatment). Steers were fed either a control diet (CON) or control diet plus 100 g∙steer^−1^∙d^−1^ supplemental microalgae meal (ALGAE; Alltech Inc., Nicholasville, KY), 100 g∙steer^−1^∙d^−1^ supplemental microalgae meal plus antioxidants (103 IU/d vitamin E and Sel-Plex; Alltech Inc.) fed throughout feeding (AOX), or 100 g∙steer^−1^∙d^−1^ supplemental microalgae meal plus antioxidants fed for the final 10 d of finishing (LATE). ^a,b,c^Within a day, means without common superscripts differ (*P* < 0.05).

As expected, day of display affected L*, a*, and b* values of steaks (*P* < 0.01; Figure 1) and was consistent with discoloration patterns seen during retail display.

There was no treatment × day interaction (*P* = 0.08) for steak L* value, but there were treatment × day interactions for steak a* and b* values, and steak surface oxy- and metmyoglobin percentages (*P* < 0.01). Therefore, treatment means for these measures were compared within each day of display. From days 0 to 4, there were no treatment differences for a* or b* (*P* > 0.08). On day 5 of display, a* values of steaks from ALGAE steers were smaller (*P* = 0.05) than steaks from AOX steers, but were similar to CON and LATE steaks (*P* > 0.14). Steaks from CON, LATE, and AOX steers had similar a* values on day 5 of display (*P* > 0.18). On day 6 of display, steaks from ALGAE steers possessed smaller a* values than steaks from AOX and LATE steers (*P* < 0.01), but values of steaks from these two treatments were not different (*P* = 0.75). Also, on this day of display, CON steak a* value did not differ from all treatments (*P* > 0.18). From day 7 throughout the remainder of the display period, steaks from ALGAE steers had reduced a* values compared with steaks from the other three treatments (*P* < 0.02). Also, AOX steak a* value did not differ (*P* = 0.06) from LATE steak a* value, but AOX steaks a had greater (*P* = 0.04) a* value than CON steaks. Finally, during this time period, the a* value of CON steaks did not differ (*P* = 0.19) from LATE steaks.

On day 5 of display, ALGAE steaks had a decreased (*P* = 0.03) b* value than AOX steaks, but did not differ from CON and LATE steaks (*P* > 0.12). Steaks from CON, AOX, and LATE steers did not possess different b* values on day 5 of display (*P* > 0.06). On day 6 of display, ALGAE steaks had a smaller b* value than AOX and LATE steaks (*P* < 0.01), but did not differ (*P* = 0.27) from CON steaks. Steaks from CON, AOX, and LATE steers did not differ in b* values (*P* > 0.08). On day 7 and throughout the rest of display, steaks from ALGAE steers possessed smaller b* values than steaks from all other treatments (*P* < 0.01). Steaks from CON steers had decreased b* values than steaks from ALGAE steers (*P* < 0.01), but did not differ (*P* = 0.14) from LATE steaks. Finally, b* values of steaks from AOX and LATE steers did not differ (*P* > 0.06).

**Figure 2.**
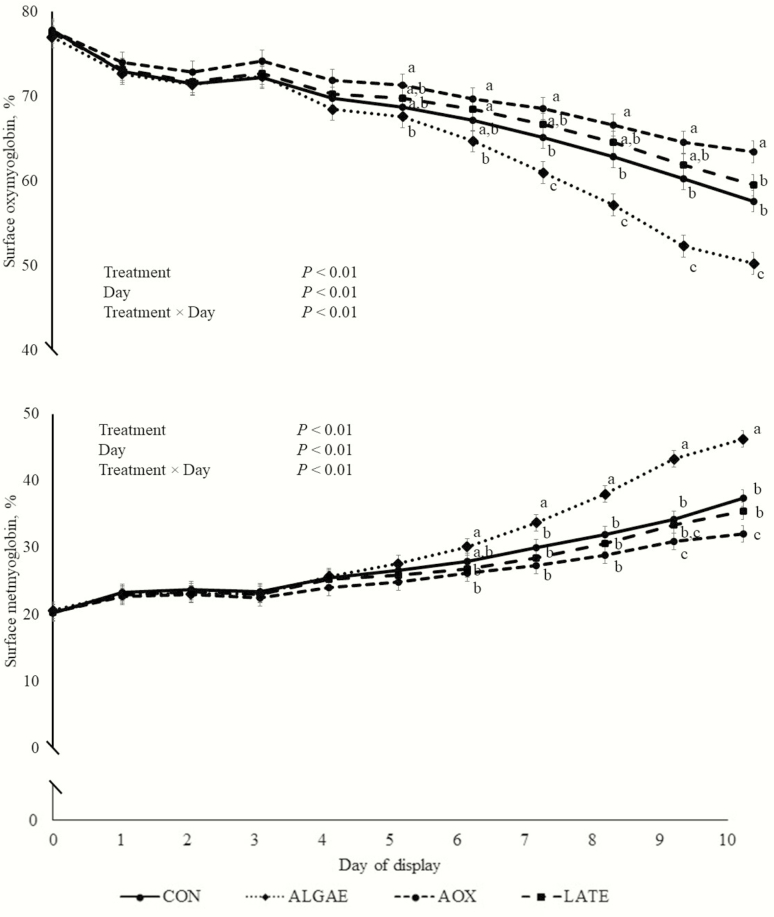
Longissimus lumborum steak surface oxymyoglobin and metmyoglobin percentages from steers fed four dietary microalgae meal and antioxidant feeding strategies (*n* = 10 per treatment). Steers were fed either a control diet (CON), supplemental DHA-rich microalgae (Alltech Inc., Nicholasville, KY) at 100 g∙steer^−1^∙d^−1^ (ALGAE), supplemental DHA-rich microalgae at 100 g∙steer^−1^∙d^−1^ and antioxidant (vitamin E 103 IU/d and Sel-Plex [Alltech Inc.]; AOX), or supplemental DHA-rich microalgae at 100 g∙steer^−1^∙d^−1^ and antioxidant (vitamin E 103 IU/d and Sel-Plex [Alltech Inc.]) for the final 10 d of finishing (LATE). ^a,b,c^Within a day, means without common superscripts differ (*P* < 0.05).

From day 0 through day 4, there were no treatment differences in steak surface oxymyoglobin percentage (*P* > 0.16; Figure 2). On day 5, ALGAE steaks had less surface oxymyoglobin (*P* = 0.03) than AOX steaks, but did not differ from CON and LATE steaks (*P* > 0.20). On day 6 of display, ALGAE steaks did not differ (*P* = 0.15) in surface oxymyoglobin compared with CON steaks, but had less surface oxymyoglobin than AOX and LATE steaks (*P* < 0.03). Steaks from AOX, LATE, and CON steers did not differ in surface oxymyoglobin percentage on this day of display (*P >* 0.13). From day 7 through day 9 of display, ALGAE steaks had less surface oxymyoglobin than the other three treatments (*P* < 0.01), AOX steaks had more (*P* < 0.04) surface oxymyoglobin than CON steaks, and LATE steaks did not differ compared with ALGAE and CON steaks (*P* > 0.27). On day 10 of display, ALGAE steaks had less oxymyoglobin than all other treatments (*P* < 0.01), and LATE and CON steaks had less oxymyoglobin than AOX steaks (*P* < 0.02), but did not differ (*P* = 0.27) from each other.

Treatments did not differ in steak surface metmyoglobin percentage through day 5 of display (*P* > 0.10). On day 6 of display, ALGAE steaks had more surface metmyoglobin than AOX and LATE steaks (*P* < 0.05), but ALGAE steaks did not differ (*P* = 0.18) from CON steaks. Steaks from AOX, LATE, and CON steers did not differ in amount of metmyoglobin on this day (*P* > 0.29). From day 7 through the remainder of display, ALGAE steaks had more metmyoglobin than steaks from the other three treatments (*P* < 0.02). From days 7 through 9, AOX steaks did not differ in amount of surface metmyoglobin compared with LATE steaks (*P* > 0.14), but AOX steaks had less (*P* = 0.04) metmyoglobin on day 10 of display. Steaks from CON and LATE did not differ in amount of surface metmyoglobin from day 7 through the end of display (*P* > 0.25).

**Figure 3.**
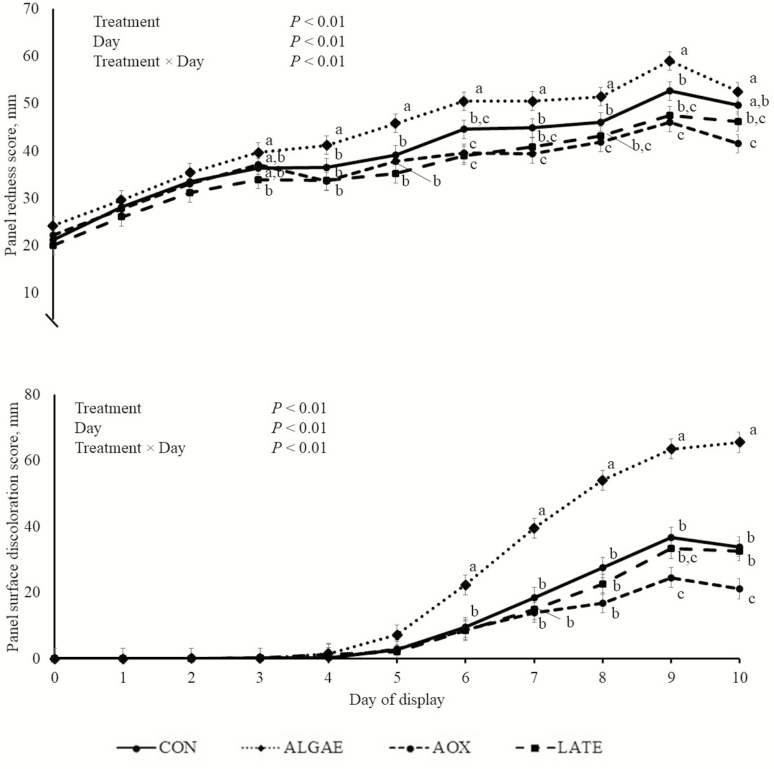
Visual panel redness and surface discoloration score of longissimus lumborum steaks from steers fed four dietary microalgae meal and antioxidant feeding strategies (*n* = 10 per treatment). Steers were fed either a control diet (CON), supplemental DHA-rich microalgae (Alltech Inc., Nicholasville, KY) at 100 g∙steer^−1^∙d^−1^ (ALGAE), supplemental DHA-rich microalgae at 100 g∙steer^−1^∙d^−1^ and antioxidant (vitamin E 103 IU/d and Sel-Plex [Alltech Inc.]; AOX), or supplemental DHA-rich microalgae at 100 g∙steer^−1^∙d^−1^ and antioxidant (vitamin E 103 IU/d and Sel-Plex [Alltech Inc.]) for the final 10 d of finishing (LATE). Utilizing continuous 100-mm line scales on paper ballots, panelists evaluated redness using six anchors points where 0 mm = 1 or light-pinkish red and 100 mm = 6 or very dark red with anchors spaced every 20 mm. Additionally, panelists assessed surface discoloration as a percentage of the longissimus muscle area also on 100-mm continuous line scales with anchors at 0 = no discoloration and 100 = 100%. ^a,b,c^Within a day, means without common superscripts differ (*P* < 0.05).

There were treatment × day interactions for visual panel redness and surface discoloration scores (*P* < 0.01; Figure 3). From days 0 to 2 of display, there were no treatment differences for visual panel redness (*P* > 0.13). On day 3 of display, ALGAE steaks did not differ in redness compared with AOX and CON steaks (*P* > 0.25), but ALGAE steaks had a greater (*P* = 0.04) redness score than LATE steaks. Steaks from CON, AOX, and LATE steers do not differ in redness scores on day 3 (*P* > 0.26). At day 4 of display, ALGAE steaks had greater redness scores than steaks from the other three treatments (*P* < 0.01), but CON, AOX, and LATE steaks did not differ (*P* > 0.31). From days 5 to 9 of display, ALGAE steaks had greater redness scores than steaks from the other three treatments (*P* < 0.01), but on day 10, ALGAE steak redness was not different (*P* = 0.31) from CON steaks. Steaks from AOX and LATE steers did not differ redness scores from day 5 until the end of display (*P* > 0.10). Additionally, from day 5 until the end of display, CON steaks redness did not differ from LATE steaks (*P* > 0.07). Steaks from AOX steers had decreased redness scores compared with CON steaks from day 5 until the end of display (*P* < 0.05).

From day 0 to 5 of display, there were no treatment differences for panel surface discoloration (*P* > 0.39). Steaks from ALGAE steers had great discoloration scores than steaks from the other three treatments from day 6 until the end of display (*P* < 0.03). From day 6 through day 8, CON, AOX, and LATE steaks did not differ in surface discoloration score (*P* > 0.08). On days 9 and 10, CON steaks had greater discoloration scores than AOX steaks (*P* < 0.04), but did not differ from LATE steaks (*P* > 0.58). Finally, on days 9 and 10 of display, AOX and LATE steaks did not differ in discoloration score (*P* > 0.06).

### Metmyoglobin-Reducing Ability and Oxygen Consumption

There were no treatment × day interactions or treatment main effects for MRA or oxygen consumption (*P* > 0.09; Table 3). As expected, MRA decreased (*P* < 0.01) with day of display. Similarly, oxygen consumption decreased with day of display (*P* < 0.01).

**Table 3.** Metmyoglobin-reducing ability and oxygen consumption of longissimus lumborum steaks from steers fed four dietary microalgae meal and antioxidant feeding strategies

	Treatment^1^					*P*-value		
	CON	ALGAE	AOX	LATE	SEM	Treatment	Day	Treatment × Day
	*n* = 10	*n* = 10	*n* = 10	*n* = 10				
Metmyoglobin-reducing ability^2^, %					2.11	0.75	<0.01	0.63
Day 0^a^	48.02	50.06	48.75	48.56				
Day 5^b^	22.67	25.64	27.95	24.95				
Day 10^c^	4.19	3.72	4.37	5.87				
Oxygen consumption^3^, %					3.79	0.15	<0.01	0.96
Day 0^a^	53.18	59.46	55.49	48.51				
Day 5^b^	11.12	19.19	16.79	12.23				
Day 10^c^	9.75	16.07	11.44	10.15				

^a,b,c^Days within analysis with different superscripts differ (*P* < 0.05). ^1^Diets consisted of a control diet (CON) or control diet plus 100 g∙steer^−1^∙d^−1^ supplemental microalgae meal (ALGAE; Alltech Inc., Nicholasville, KY), 100 g∙steer^−1^∙d^−1^ supplemental microalgae meal plus antioxidants (103 IU/d vitamin E and Sel-Plex; Alltech Inc.) fed throughout feeding (AOX), and 100 g∙steer^−1^∙d^−1^ supplemental microalgae meal plus antioxidants fed for the final 10 d of finishing (LATE). ^2^Metmyoglobin reducing ability was calculated as follows: (observed decreased in metmyoglobin concentration/initial metmyoglobin concentration) × 100. ^3^Oxygen consumption was calculated as follows: (observed decreased in oxymyoglobin concentration/initial oxymyoglobin concentration) × 100.

### Sensory Analysis

There were no treatment differences observed for initial or sustained juiciness of steaks (*P* > 0.21; Table 4). Also, treatments did not affect myofibrillar tenderness, connective tissue amount, and overall tenderness of LL steaks (*P* > 0.70). Treatments did not affect beef-flavor identity or beef-flavor intensity (*P* > 0.15), but off-flavor intensity was affected (*P* < 0.01) by treatment. Steaks from CON steers had less off-flavor intensity than ALGAE and AOX steaks (*P* < 0.03), but did not differ (*P* = 0.07) in intensity when compared with LATE steaks. Steaks from all three microalgae treatments did not differ in off-flavor intensity scores (*P* > 0.10).

**Table 4.** Trained sensory panel evaluation of longissimus lumborum steaks from steers fed four dietary microalgae meal and antioxidant feeding strategies

	Treatment^1^					
	CON	ALGAE	AOX	LATE		
	*n* = 10	*n* = 10	*n* = 10	*n* = 10	SEM	*P*-value
Attribute^2^						
Initial juiciness	65.34	60.94	65.98	67.28	2.66	0.38
Sustained juiciness	56.25	49.09	55.15	57.70	3.00	0.21
Myofibrillar tenderness	65.89	64.76	67.62	68.47	3.28	0.85
Connective tissue	20.16	18.70	16.46	19.28	2.35	0.70
Overall tenderness	60.60	60.15	63.12	62.78	3.40	0.90
Beef flavor identity	60.59	54.17	57.88	55.93	2.00	0.15
Beef flavor intensity	45.80	40.26	43.54	40.10	2.53	0.33
Off-flavor intensity	1.68^a^	4.65^b^	6.82^b^	4.11^a,b^	0.97	<0.01

^a,b,c^Within a row, means without a common superscript differ (*P* < 0.05). ^1^Diets consisted of a control diet (CON) or control diet plus 100 g∙steer^−1^∙d^−1^ supplemental microalgae meal (ALGAE; Alltech Inc., Nicholasville, KY), 100 g∙steer^−1^∙d^−1^ supplemental microalgae meal plus antioxidants (103 IU/d vitamin E and Sel-Plex; Alltech Inc.) fed throughout feeding (AOX), and 100 g∙steer^−1^∙d^−1^ supplemental microalgae meal plus antioxidants fed for the final 10 d of finishing (LATE). ^2^0 = extremely dry, extremely dry, extremely tough, none, extremely tough, extremely unbeef-life, extremely bland, and extremely bland; 100 = extremely juicy, extremely juicy, extremely tender, abundant, extremely tender, extremely beef-like, extremely intense, and extremely intense.

## DISCUSSION

Numerous studies in the literature documented that feeding linseed/flaxseed and marine sources increased the omega-3 fatty acid content of meat (for review see Burnett et al., 2020). Givens et al. (2006) concluded that marine sources had the greatest ability to increase DHA and EPA meat content; however, when the omega-3 content of meat is elevated, meat discoloration accelerates during display. Color saturation is utilized as an indirect measure of metmyoglobin formation. In lamb, color saturation of chops was reduced (more metmyoglobin formed) when marine algae or fish oil was supplemented compared with linseed oil supplementation (Nute et al., 2007). In contrast, Vatansever et al. (2000) reported no differences in color saturation values of LL steaks from steers fed linseed (flaxseed) and linseed/fish oil, indicating similar amounts of metmyoglobin formed. These data could indicate a species or omega-3 source response.

Phelps et al. (2016a) demonstrated that feeding up to 150 g∙steer^−1^∙d^−1^ of the same microalgae product utilized in the current study quadratically increased DHA and EPA content. The increase in omega-3 content subsequently caused LL steak a* values to decrease and surface metmyoglobin to increase quadratically. Additionally, ground beef produced from knuckles and fat trim from the same heifers showed a linear decrease in a* value and increase in surface metmyoglobin accumulation during display (Phelps et al., 2016b). Although the current study has no available fatty acid profile data, the microalgae supplement was fed for 44 less days, and all diets had a flaxseed component, the detrimental reduction in color characteristics stimulated by microalgae was still demonstrated in the ALGAE steaks. Additionally, carcass data indicated that the color-stability differences could not be due to intramuscular adipose content differences, as each treatment did not differ in marbling score. Unlike the study by Phelps et al. (2016a), which saw immediate negative color effects in steaks from microalgae fed heifers, it took until day 7 of display for ALGAE steaks to have color differences compared with CON steaks. This differential response in color deterioration was most likely due to the reduced time for the feeding trial and possible inclusion of flaxseed in the base diet; however, the model utilized is a valid model for demonstrating the negative effects of dietary microalgae on color stability because panelists rated ALGAE steaks as being darker red on day 3 of display and possessing more discoloration on day 6.

Supplementation of vitamin E, which is the major lipid-soluble antioxidant in animal tissues, can delay oxidation of beef (Wood and Enser, 1997). Many researchers have demonstrated supplementing vitamin E over the nutritional requirement improves steak redness and stability of color during display (Faustman et al., 1989; Arnold et al., 1993; Lynch et al., 1999). Researchers also investigated selenium as an antioxidant source in meat systems; however, a bulk of the literature indicated it does not affect meat color (O’Grady et al., 2001; Taylor et al., 2008; Jose et al., 2010). In the current study, additional dietary vitamin E and selenium-yeast to diets containing microalgae meal improved meat color characteristics over CON and ALGAE steaks, especially at the end of display. Specifically, a* values were greater, surface oxymyoglobin percentage was greater, and surface metmyoglobin percentage were reduced when vitamin E and selenium were added to the diet. Additionally, panelists also visually detected this same trend with AOX steaks having 45% less surface discoloration than ALGAE steaks. The pattern of color responses seems to indicate that supplementing the two antioxidants during the entire feeding period had a greater effect on maintaining color-stability than just adding them the final 10 d of feeding.

Numerous studies indicated that vitamin E improved meat color characteristics when beef omega-3 content is elevated with linseed/flaxseed. Alberti et al. (2014) found no negative effect on L* value of steaks from Pirenaica bulls supplemented linseed when vitamin E was added to diets. Juárez et al. (2012) reported that supplementing vitamin E at levels greater than 451 IU/d to 10% flaxseed diets prevented a reduction in steak lightness commonly observed and reduced surface metmyoglobin accumulation. O’Grady et al. (2001) found that when organic selenium and 300 IU of vitamin E were supplemented, more oxymyoglobin was present on minced LL at the end of 14-d display in 80% oxygen-modified atmosphere packaging. Much like this study, because vitamin E and selenium were supplemented together, it is impossible to discern which contributed more to the improved color in the current study.

In the current study, steer-feeding treatment did not affect OCR and MRA. McKenna et al. (2005) concluded that beef color stability is controlled by the muscle-specific relationship between OCR and MRA. The literature indicates muscles with greater OCR and reduced MRA possessed poorer color stability (O’Keeffe and Hood, 1982; Ledward, 1985; McKenna et al., 2005). Although it is logical that dietary treatment in the current study would not affect OCR, MRA could have been affected. In muscles with elevated omega-3 fatty acids, lipid oxidation is a major catalyzer of metmyoglobin formation. Specifically, the products of oxidation accelerate myoglobin oxidation (Faustman et al., 1999) and reduce MRA. Phelps et al. (2016a) demonstrated that feeding microalgae increased lipid oxidation, but did not measure MRA. Mitsumoto et al. (1991) hypothesized that vitamin E indirectly maintained metmyoglobin-reducing activity by interacting with free radicals and preventing lipid oxidation. Lee et al. (2003) also found that vitamin E increased total reducing ability and stabilized LL color of steaks from Hanwoo cattle. In agreement with the current study, Chan et al. (1998) reported that vitamin E improved color stability but did not affect aerobic metmyoglobin-reducing ability. Therefore, these findings could indicate that detecting the positive influence antioxidants elicit on MRA may be dependent on the assay used to quantify MRA.

In addition to lipid oxidation products catalyzing undesirable color formation, these products also produce off-flavor compounds. In lamb, supplementing marine algae and a protected oil containing linoleic and linolenic acid increased abnormal lamb flavors of lamb chops compared with supplementing linseed oil alone (Nute et al., 2007). Juárez et al. (2012) reported that adding supplemental vitamin E to 10% flaxseed diets caused no differences in sensory panel tenderness, juiciness, or flavor. Previously, Phelps et al. (2016a,b) found that feeding increased levels of microalgae meal to heifers caused a quadratic increase in panelists detection of steak and ground beef off-flavors. In the current study, supplementing microalgae for 44 d increased product off-flavors regardless of antioxidant supplementation; however, because the values span less than 10% of the scale, it is possible that these differences could not be detected by the consumer. It is important to acknowledge that long-chain PUFA are overall more susceptible to oxidation, and increasing their level within the products is going to increase oxidation overall (Jacobsen et al., 2008), which is why panelists detected small levels of off-flavors. Additionally, most of the off-flavors noted by the panelists in the current study were classified as “fishy.” This could indicate that the microalgae is depositing a compound in the fat unique to marine species and why the treatments fed the microalgae for a longer period (ALGAE and AOX) had greater off-flavor scores than CON steaks.

## CONCLUSION

The color stability of LL steaks from steers receiving dietary microalgae was improved when the antioxidants, vitamin E, and selenium-yeast were supplemented. It is impossible to discern whether vitamin E or selenium-yeast had a bigger impact on color. Further research is needed to compare differences in steak color stability when cattle are supplemented vitamin E or selenium-yeast in diets containing microalgae. Although antioxidants had noticeable differences in color stability, they were unable to reduce trained panelists off-flavor detection. Even though these differences were minute, further research is necessary to examine whether these off-flavors would be detectable by consumers.

## References

[CIT0001] Alberti P., Beriain M. J., Ripoll G., Sarriés V., Panea B., Mendizabal J. A., Purroy A., Olleta J. L., and Sañudo C. 2014 Effect of including linseed in a concentrate fed to young bulls on intramuscular fatty acids and beef color. Meat Sci. 96:1258–1265. doi:10.1016/j.meatsci.2013.11.009 24334048

[CIT0002] American Meat Science Association (AMSA). 2012 Meat color measurement guidelines. AMSA, Champaign, IL.

[CIT0003] American Meat Science Association (AMSA). 2015 Research guidelines for cookery, sensory evaluation, and instrumental tenderness measurements of meat. 2nd ed. AMSA, Champaign, IL.

[CIT0004] Arnold R. N., Arp S. C., Scheller K. K., Williams S. N., and Schaefer D. M. 1993 Tissue equilibration and subcellular distribution of vitamin E relative to myoglobin and lipid oxidation in displayed beef. J. Anim. Sci. 71:105–118. doi:10.2527/1993.711105x 8454531

[CIT0005] Burnett D. D., Legako J. F., Phelps K. J., and Gonzalez J. M. 2020 Biology, strategies, and fresh meat consequences of manipulating the fatty acid composition of meat. J. Anim. Sci. 98:1–12. doi:10.1093/jas/skaa033 PMC7036598 31999826

[CIT0006] Calder P. C 2014 Very long chain omega-3 (n-3) fatty acids and human health. Eur. J. Lipid. Sci. Technol. 116:1280–1300. doi:10.1002/ejlt.201400025

[CIT0007] Chan W. K., Faustman C., Velasquez-Pereira J., McDowell L. R., and Batra T. R. 1998 Effects of alpha-tocopherol on metmyoglobin formation and reduction in beef from cattle fed soybean or cottonseed meal diets. J. Anim. Sci. 76:1421–1426. doi:10.2527/1998.7651421x 9621948

[CIT0008] Cooper S. L., Sinclair L. A., Wilkinson R. G., Hallett K. G., Enser M., and Wood J. D. 2004 Manipulation of the n-3 polyunsaturated fatty acid content of muscle and adipose tissue in lambs. J. Anim. Sci. 82:1461–1470. doi:10.2527/2004.8251461x 15144087

[CIT0009] Daly C. M., Moloney A. P., and Monahan F. J. 2007 Lipid and colour stability of beef from grazing heifers supplemented with sunflower oil alone or with fish oil. Meat Sci. 77:634–642. doi:10.1016/j.meatsci.2007.05.016 22061952

[CIT0010] Faustman C., Cassens R. G., Schaefer D. M., Buege D., Williams S. N., and Scheller K. K. 1989 Improvement of pigment and lipid stability in Holstein steer beef by dietary supplementation with vitamin E. J. Food Sci. 54:858–862. doi:10.1111/j.1365-2621.1989.tb07899.x

[CIT0011] Faustman C., Liebler D. C., McClure T. D., and Sun Q. 1999 Alpha,beta-unsaturated aldehydes accelerate oxymyoglobin oxidation. J. Agric. Food Chem. 47:3140–3144. doi:10.1021/jf990016c 10552621

[CIT0012] Givens D. I., Kliem K. E., and Gibbs R. A. 2006 The role of meat as a source of n-3 polyunsaturated fatty acids in the human diet. Meat Sci. 74:209–218. doi:10.1016/j.meatsci.2006.04.008 22062730

[CIT0013] Gobert M., Gruffat D., Habeanu M., Parafita E., Bauchart D., and Durand D. 2010 Plant extracts combined with vitamin E in PUFA-rich diets of cull cows protect processed beef against lipid oxidation. Meat Sci. 85:676–683. doi:10.1016/j.meatsci.2010.03.024 20416810

[CIT0014] Jacobsen C., Bruni M., Nielsen N. S., and Meyer A. S. 2008 Antioxidant strategies for preventing oxidative flavour deterioration of foods enriched with n-3 polyunsaturated lipids: A comparative evaluation Trend. Food Sci. Tech. 19:76–93. doi:10.1016/j.jpgs.2007.08.001

[CIT0015] Jose C. G., Jacob R. H., Gardner G. E., Pethick D. W., and Liu S. M. 2010 Selenium supplementation and increased muscle glutathione concentration do not improve the color stability of lamb meat. J. Agric. Food Chem. 58:7389–7393. doi:10.1021/jf100191k 20491438

[CIT0016] Juárez M., Dugan M. E., Aldai N., Basarab J. A., Baron V. S., McAllister T. A., and Aalhus J. L. 2012 Beef quality attributes as affected by increasing the intramuscular levels of vitamin E and omega-3 fatty acids. Meat Sci. 90:764–769. doi:10.1016/j.meatsci.2011.11.010 22115728

[CIT0018] Krzywicki K. 1979 Assessment of relative content of myoglobin, oxymyoglobin, and metmyoglobin at the surface of beef. Meat Sci. 3:1–10. doi:10.1016/0309-1740(79)90019-6 22055195

[CIT0019] Ledward D. A 1985 Post slaughter influences on the formation of metmyoglobin in beef muscle. Meat Sci. 15:149–171. doi:10.1016/0309-1740(85)90034-8 22054503

[CIT0020] Lee S. K., Kim Y. S., Liang C. Y., and Song Y. H. 2003 Effects of dietary vitamin E supplementation on color stability, lipid oxidation and reducing ability of Hanwoo (Korean cattle) beef during retail display. Asain-Aust. J. Anim. Sci. 16:1529–1534. doi:10.5713/ajas.2003.1529

[CIT0021] Lucherk L. W., O’Quinn T. G., Legako J. F., Rathmann R. J., Brooks J. C., and Miller M. F. 2016 Consumer and trained panel evaluation of beef strip steaks of varying marbling and enhancement levels cooked to three degrees of doneness. Meat Sci. 122:145–154. doi:10.1016/j.meatsci.2016.08.005 27544884

[CIT0022] Luo H., and Huang C. L. 2012 Demand for healthful and unhealthful foods: Do prices matter on obesity? J. Agribuis. 30:69–86. doi:10.22004/ag.econ.260190

[CIT0023] Lynch M. P., Kerry J. P., Buckley D. J., Faustman C., and Morrissey P. A. 1999 Effect of dietary vitamin E supplementation on the colour and lipid stability of fresh, frozen and vacuum-packaged beef. Meat Sci. 52:95–99. doi:10.1016/s0309-1740(98)00153-3 22062148

[CIT0024] McKenna D. R., Mies P. D., Baird B. E., Pfeiffer K. D., Ellebracht J. W., and Savell J. W. 2005 Biochemical and physical factors affecting discoloration characteristics of 19 bovine muscles. Meat Sci. 70:665–682. doi:10.1016/j.meatsci.2005.02.016 22063894

[CIT0025] Mitsumoto M., Cassens R. G., Schaefer D. M., Arnold R. N., and Scheller K. K. 1991 Improvement of color and lipid stability in beef longissimus with dietary vitamin E and vitamin C dip treatment. J. Food Sci. 56:1489–1492. doi:10.1111/j.1365-2621.1991.tb08622.x

[CIT0026] Nute G. R., Richardson R. I., Wood J. D., Hughes S. I., Wilkinson R. G., Cooper S. L., and Sinclair L. A. 2007 Effect of dietary oil source on the flavour and the colour and lipid stability of lamb meat. Meat Sci. 77:547–555. doi:10.1016/j.meatsci.2007.05.003 22061940

[CIT0027] O’Grady M. N., Monahan F. J., Fallon R. J., and Allen P. 2001 Effects of dietary supplementation with vitamin E and organic selenium on the oxidative stability of beef. J. Anim. Sci. 79:2827–2834. doi:10.2527/2001.79112827x 11768111

[CIT0028] O’Keeffe M., and Hood D. E. 1982 Biochemical factors influencing metmyoglobin formation on beef from muscles of differing color stability. Meat Sci. 7:209–228. doi:10.1016/0309-1740(82)90087-0 22055235

[CIT0029] Phelps K. J., Drouillard J. S., Jennings J. S., Depenbusch B. E., Van Bibber-Krueger C. L., Miller K. A., Vaughn M. A., Burnett D. D., Ebarb S. M., Houser T. A., et al. 2014 Effects of the programmed nutrition beef program on meat quality characteristics. J. Anim. Sci. 92:1780–1791. doi:10.2527/jas.2013-7231 24492560

[CIT0030] Phelps K. J., Drouillard J. S., O’Quinn T. G., Burnett D. D., Blackmon T. L., Axman J. E., Van Bibber-Krueger C. L., and Gonzalez J. M. 2016a Feeding microalgae meal (All-G Rich; CCAP 4067/2) to beef heifers. I: Effects on ground beef color and palatability. J. Anim. Sci. 94:4016–4029. doi:10.2527/jas.2016-0488 27898910

[CIT0031] Phelps K. J., Drouillard J. S., O’Quinn T. G., Burnett D. D., Blackmon T. L., Axtman J., Van Bibber-Krueger C. L., and Gonzalez J. M. 2016b Feeding microalgae meal (All-G Rich™, Schizochytrium limacinum CCAP 4087/2) to beef heifers II: Effects on ground beef palatability and color. J. Anim. Sci. 94:4030–4039. doi:10.2527/jas2016-0488 27898910

[CIT0032] Rossi C. A. S., Compiani R., Baldi G., Bernardi C. E. M., Muraro M., Marden J. P., and Dell’Orto V. 2015 The effect of different selenium source during the finishing phase on beef quality. J. Anim. Feed Sci. 24:93–99. doi:10.22358/jafs/65633/2015

[CIT0033] Ruxton C. H., Reed S. C., Simpson M. J., and Millington K. J. 2004 The health benefits of omega-3 polyunsaturated fatty acids: A review of the evidence. J. Hum. Nutr. Diet. 17:449–459. doi:10.1111/j.1365-277X.2004.00552.x 15357699

[CIT0034] Saunders E. F., Reider A., Singh G., Gelenberg A. J., and Rapoport S. I. 2015 Low unesterified:esterified eicosapentaenoic acid (EPA) plasma concentration ratio is associated with bipolar disorder episodes, and omega-3 plasma concentrations are altered by treatment. Bipolar Disord. 17:729–742. doi:10.1111/bdi.12337 26424416 PMC4623957

[CIT0035] Taylor J. B., Marchello M. J., Finley J. W., Neville T. L., Combs G. F., and Caton J. S. 2008 Nutritive value and display-life attributes of selenium-enriched beef-muscle foods. J. Food Compost. Anal. 21:183–186. doi:10.1016/j.jfca.2007.08.001

[CIT0036] Van Bibber-Krueger C. L., Collins A. M., Phelps K. J., O’Quinn T. G., Houser T. A., Turner K. K., and Gonzalez J. M. 2020 Effects of quality grade and intramuscular location on beef semitendinosus muscle fiber characteristics, NADH content, and color stability. J. Anim. Sci. 98:1–11. doi:10.1093/jas/skaa078 PMC7141495 32157294

[CIT0037] Vatansever L., Kurt E., Enser M., Nute G. R., Scollan N. D., Wood J. D., and Richardson R. I. 2000 Shelf life and eating quality of beef from cattle of different breeds given diets differing in n-3 polyunsaturated fatty acid composition. Animal 71:471–482.

[CIT0038] Vierck K. R., Gonzalez J. M., Houser T. A., Boyle E. A. E., and O’Quinn T. G. 2018 Marbling texture’s effects on beef palatability. Meat Muscle Biol. 2:1–12. doi:10.22175/mmb2017.10.0052

[CIT0039] Wistuba T. J., Kegley E. B., and Apple J. K. 2006 Influence of fish oil in finishing diets on growth performance, carcass characteristics, and sensory evaluation of cattle. J. Anim. Sci. 84:902–909. doi:10.2527/2006.844902x 16543568

[CIT0040] Wood J. D., and Enser M. 1997 Factors influencing fatty acids in meat and the role of antioxidants in improving meat quality. Br. J. Nutr. 78(Suppl. 1):S49–S60. doi:10.1079/bjn19970134 9292774

[CIT0041] Woods V. B., and Fearon A. M. 2009 Dietary sources of unsaturated fatty acids for animals and their transfer into meat, milk and eggs: A review. Livest. Sci. 126:1–20. doi:10.1016/j.livesci.2009.07.002

[CIT0042] Yang A., Lanari M. C., Brewster M., and Tume R. K. 2002 Lipid stability and meat colour of beef from pasture- and grain-fed cattle with or without vitamin E supplement. Meat Sci. 60:41–50. doi:10.1016/s0309-1740(01)00103-6 22063104

